# Comparative Transcriptome and Chloroplast Genome Analyses of Two Related *Dipteronia* Species

**DOI:** 10.3389/fpls.2016.01512

**Published:** 2016-10-13

**Authors:** Tao Zhou, Chen Chen, Yue Wei, Yongxia Chang, Guoqing Bai, Zhonghu Li, Nazish Kanwal, Guifang Zhao

**Affiliations:** ^1^Key Laboratory of Resource Biology and Biotechnology in Western China (Ministry of Education), College of Life Sciences, Northwest UniversityXi'an, China; ^2^Shaanxi Engineering Research Centre for Conservation and Utilization of Botanical Resources, Xi'an Botanical Garden of Shaanxi ProvinceXi'an, China

**Keywords:** *Dipteronia*, transcriptome, positive selection, purifying selection, chloroplast genome, phylogenetic relationship

## Abstract

*Dipteronia* (order Sapindales) is an endangered genus endemic to China and has two living species, *D*.sinensis and *D. dyeriana*. The plants are closely related to the genus *Acer*, which is also classified in the order Sapindales. Evolutionary studies on *Dipteronia* have been hindered by the paucity of information on their genomes and plastids. Here, we used next generation sequencing to characterize the transcriptomes and complete chloroplast genomes of both *Dipteronia* species. A comparison of the transcriptomes of both species identified a total of 7814 orthologs. Estimation of selection pressures using Ka/Ks ratios showed that only 30 of 5435 orthologous pairs had a ratio significantly >1, i.e., showing positive selection. However, 4041 orthologs had a Ka/Ks < 0.5 (*p* < 0.05), suggesting that most genes had likely undergone purifying selection. Based on orthologous unigenes, 314 single copy nuclear genes (SCNGs) were identified. Through a combination of *de novo* and reference guided assembly, plastid genomes were obtained; that of *D. sinensis* was 157,080 bp and that of *D. dyeriana* was 157,071 bp. Both plastid genomes encoded 87 protein coding genes, 40 tRNAs, and 8 rRNAs; no significant differences were detected in the size, gene content, and organization of the two plastomes. We used the whole chloroplast genomes to determine the phylogeny of *D. sinensis* and *D. dyeriana* and confirmed that the two species were highly divergent. Overall, our study provides comprehensive transcriptomic and chloroplast genomic resources, which will be valuable for future evolutionary studies of *Dipteronia*.

## Introduction

*Dipteronia* Oliver (order Sapindales) is an endangered genus endemic to China; it has two living species, *D. sinensis* Oliver and *D. dyeriana* Henry, and is a sister genus of *Acer* (Peng and Thomas, 2008). The genus *Dipteronia* has been documented in the fossil record with specimens found in Tertiary sediments in North America (McClain and Manchester, 2001). Both extant species are perennial woody plants with different natural ranges; *D. sinensis* occupies a relatively extensive range in central and southwestern China, while *D. dyeriana* is located in a limited area of Yunnan province. The latter species is grown as an ornamental species and for oil. Although the two species of *Dipteronia* are allopatric at the present time, they share some morphological similarities such as leaf shape and fruit characteristics. However, comparatively little is known of the genetic differentiation of the two species or their evolutionary dynamics.

As relic species of the Tertiary period, both species of *Dipteronia* have experienced long complex evolutionary histories to result in their current distributional status. Previous research based on analyses of chloroplast simple sequence repeats (cpSSRs) and amplified fragment length polymorphisms (AFLPs) revealed that significant genetic differences are present between *D. sinensis* and *D. dyeriana*; these analyses also suggested that the populations of *Dipteronia* may have suffered a genetic bottleneck (Yang et al., 2007, 2008). However, a clear understanding of the causes of their genetic divergence and speciation has still not been achieved; this is largely because of the lack of genomic resources. To date, comprehensive genome sequences and complete chloroplast genomes have not been described for either species. Nor has any attempt been made at comparative transcriptomics to identify possible causes of genome divergence and selection in these two species.

Next-generation sequencing (NGS) has greatly advanced our ability to obtain genome resources in non-model species. Transcriptome sequencing (RNA-seq) offers both a convenient means of rapidly obtaining information on expressed genomic regions and also provides an opportunity to resolve comparative genomic-level problems for non-model organisms (Logacheva et al., 2011; Zhang L. et al., 2013). With the advent of NGS, transcriptome sequencing has become more effective. Transcriptome sequencing also provides an alternative method for whole-genome sequencing for use in analyzing adaptive evolution and genetic divergence (Zhang L. et al., 2013; Chen et al., 2015; Mu et al., 2015). For closely related species, comparative transcriptome analyses can not only provide useful genomic resources, such as SSRs and single copy nuclear gene (SCNG) markers, but can also provide insights into speciation and adaptive evolution.

In plants, the chloroplast genome is more conserved than the nuclear genome; it usually has a circular structure of a pair of inverted repeat (IR) regions separated by large single-copy (LSC) and small single-copy (SSC) regions (Bendich, 2004). Because of its conserved nature, many plastid molecular markers have been used to infer phylogeographic history as well as to resolve the phylogenetic relationships of different species. The availability of NGS technology has enabled the generation of large amounts of sequence data at relatively low cost. Thus, it is comparatively simple to obtain comprehensive chloroplast sequences for plant species with this new technology. Sequencing of the complete chloroplast genome has been used in phylogenetic analyses and has proved effective in clarifying difficult phylogenetic relationships (Ma et al., 2014; Carbonell-Caballero et al., 2015). However, until now, only two chloroplast genomes have been reported for the Aceraceae (Yang J. B. et al., 2014; Li Z. H. et al., 2015). Thus, the present study on the chloroplast genomes of *Dipteronia* will provide valuable plastid resources to resolve phylogenetic relationships in *Acer* and *Dipteronia*. Furthermore, the chloroplast genome data will aid development of plastid genetic markers for phylogeographic research in *Dipteronia*.

In the present study, we compared the transcriptomes and chloroplast genomes of the two *Dipteronia* species. We also carried out pairwise comparisons of orthologous sequences from these species to identify candidate genes under positive selection. Our sequencing analysis of the transcriptomes identified a large number of single-copy nuclear gene markers from both species. Additionally, we used the information on the chloroplast genomes to analyze the phylogenetic relationships of species within the order Sapindales. Overall, our study provides new insights into the evolutionary history of the two *Dipteronia* species and has produced resources for further evolutionary studies on *Dipteronia* and related species in the Aceraceae.

## Materials and methods

### Transcriptome sequence datasets, *De novo* assembly, gene expression levels, and functional annotation

Two transcriptome datasets (SRR2127986/SRR2127991) from a previous study (Zhou et al., 2016) were used for the present comparative transcriptome analysis. Before assembly, the raw reads were filtered to obtain high-quality clean reads by removing adapters, low-quality sequences (reads with unknown bases “N”), and reads with more than 20% low-quality bases (quality value ≤ 10). High quality reads were assembled as transcripts using Trinity (r2013-02-25) with default parameters (Grabherr et al., 2011). After assembly, the resultant transcripts were processed by CD-HIT version 4.6 with a sequence identity threshold of 0.95 to remove redundancies (Li and Godzik, 2006). We used RSEM-1.2.29 software (Li and Dewey, 2011) to estimate gene expression levels in each species. First, the clean reads of each species were mapped back onto the transcripts to obtain the read count values of all genes. Then we calculated the fragments per kilobase of transcript per million mapped reads (FPKM), which is the most commonly used method to estimate gene expression levels (Trapnell et al., 2010). For evaluating the function of *D. sinensis* and *D. dyeriana* transcriptome sequences, we separately aligned the unigene sequences of these two species with public protein databases such as the NCBI non-redundant protein database (Nr), Cluster of Orthologous Group (COG), Swiss-Prot, and the Kyoto Encyclopedia of Genes and Genomes (KEGG) pathway database using blastx with an *E*-value threshold of 1E-5. Gene ontology (GO) annotation was performed by Blast2GO software with a cut-off *E*-value of 1E-5 and then plotted with functional classification using Web Gene Ontology Annotation Plot (WEGO) (Conesa et al., 2005; Ye et al., 2006).

### Identification of orthologous genes in *D. sinensis* and *D. dyeriana*, estimation of substitution rates, and mining of single copy nuclear genes

Open reading frames (ORF) of unigene sequences were predicted by the Getorf program with a minimum length of 150 amino acids (Rice et al., 2000). The predicted coding DNA sequence regions of the *D. sinensis* and *D. dyeriana* transcriptomes were then used to identify orthologous groups between the two species. OrthoMCL v2.0.9, based on a protein similarity graph method (Li et al., 2003), was employed to retrieve the groups of homologous protein coding genes with the default parameters. InParanoid 7 was also used to search the orthologous groups with the genome of *Theobroma cacao* as an outgroup (Ostlund et al., 2010). Finally, we compared the results from both methods and orthologs shared between the two methods were retrieved as the orthologous genes of two species. The remaining protein coding genes that could not be assigned to orthologous groups were considered as species-specific expressed genes. The obtained orthologous pairs were aligned and formatted by ParaAT1.0 with default parameters (Zhang Z. et al., 2012). The nonsynonymous (Ka), synonymous (Ks), and Ka/Ks values were calculated using KaKs_Calculator v. 1.2 based on the YN algorithm (Zhang et al., 2006) and Fisher's exact test was performed to justify the validity of the Ka and Ks values. For the purpose of finding SCNGs in *Dipteronia*, the 959 APVO genes (959 SCNGs shared by *Arabidopsis, Populus, Vitis*, and *Oryza*) were used for our analysis (Duarte et al., 2010). We retrieved the protein sequences encoded by the APVO genes from the TAIR10 database and then queried these sequences against the orthologous genes of *D. sinensis* and *D. dyeriana* using BLASTP with a threshold *E*-value of 1E-10. All the queries with hits were considered to be SCNGs in the *Dipteronia* species.

### The chloroplast genome sequencing, assembly, and annotation of *D. sinensis* and *D. dyeriana*

Total genomic DNA was isolated from leaf tissues using the modified CTAB method (Doyle, 1987). The DNA library was constructed using TruSeq DNA sample preparation kits and then a paired-end library with insert sizes of 200 bp was sequenced using Illumina HiSeq™ 2500 with the average read length of 125 bp. In order to conduct comparative chloroplast genome analyses of two *Dipteronia* species, the raw Illumina sequencing reads of *D. sinensis* from our previous study (Zhou et al., 2015) were retrieved in the present study. Illumina raw reads were first quality trimmed using NGS QC Toolkit_v2.3.3 with default cut-off values (Patel and Jain, 2012). After trimming of low quality reads and adapters, the clean reads were assembled using MIRA 4.0.2 (Chevreux et al., 2004) with the chloroplast genome of *Acer buergerianum* subsp. *ningpoense* (Yang J. B. et al., 2014) as a reference (parameters: job = genome, mapping, accurate; technology = solexa; segment_placement = FR). Subsequently, the resultant contigs were further assembled using a baiting and iteration method based on Perl script MITObim_1.8.pl (Hahn et al., 2013). After assembly, the obtained contigs were ordered with the reference chloroplast genome of *A. buergerianum* subsp. *Ningpoense*. The gaps were filled by realignment of input reads using Geneious R8 v 8.0.2 (Biomatters Ltd., Auckland, New Zealand) and some ambiguous regions with low coverage were confirmed by PCR-based Sanger sequencing using primers designed for gap-flanking regions (Table S9). Eventually, the complete chloroplast genome was annotated by the online software DOGMA (Wyman et al., 2004) with default parameters and manual adjustment of the start and stop codons in Geneious R8 v 8.0.2. The annotated GenBank files were used to draw circular plastid genome maps with the online program OrganellarGenome DRAW (OGDRAW) (Lohse et al., 2013).

### Repeat structure and sequence divergence of chloroplast genomes

Dispersed and palindromic repeats in each chloroplast genome were identified using REPuter with a minimum repeat size of 30 bp and a sequence identity >90% (Kurtz et al., 2001). The Tandem Repeats Finder program was used to identify tandem repeat sequences with the following parameters: 2 for alignment parameters match, 7 for mismatch and indel, respectively (Benson, 1999). SSR loci in both chloroplast genomes were detected using MISA with the SSR identification parameters of ten for mono, five for di-, four for tri-, and three for tetra-, penta, and hexa-nucleotide motifs. Construction of multiple alignments of complete cpDNA sequences was carried out by the mVISTA comparative genomics tool with the annotation of *A. buergerianum* subsp. *Ningpoense* as reference (Frazer et al., 2004). The percentages of variable characters for each coding and noncoding regions were calculated as described in a previous study of Poaceae species (Zhang et al., 2011). In order to detect whether selective pressure exists for plastid genes, we calculated the nonsynonymous (Ka), synonymous (Ks), and Ka/Ks values of each protein coding gene in the two chloroplast genomes.

### Phylogenetic analyses

The phylogeny of the *Dipteronia* species was investigated using the complete plastid genomes of species in the order Sapindales, including *A. buergerianum* subsp. *Ningpoense* (KF753631), *Acer morrisonense* (KT970611), *Sapindus mukorossi* (KM454982), *Citrus aurantifolia* (NC_024929), *Citrus sinensis* (NC_008334), *Azadirachta indica* (NC_023792), and *Zanthoxylum piperitum* (NC_0279390); these sequences were downloaded as ingroup taxa. *Populus trichocarpa* (NC_009143) and *T. cacao* (HQ244500) were used as outgroup taxa. The complete chloroplast genomes with one IR region removed were aligned by MAFFT v7.017 software with default parameters (Katoh and Standley, 2013) and then the sequences were manually adjusted using ClustalX (Larkin et al., 2007). The choice of substitution model for each partition was primarily determined using Modeltest 3.7 (Posada and Crandall, 1998) with the Akaike information criterion (AIC) (Posada and Buckley, 2004). Phylogenetic analysis was conducted based on the maximum likelihood (ML) method using RAxML version v 7.2.8 (Stamatakis, 2006). The ML tree was constructed with a combined rapid bootstrap of 1000 replicates and a search for the best tree in a single run under the GTR + G model. In parallel, phylogeny was also inferred from the plastid genomes using MrBayes v 3.1.2 (Ronquist and Huelsenbeck, 2003) with the TVM + I +G model. The Markov chain Monte Carlo (MCMC) algorithm was run for one million generations with trees sampled very 100 generations. Convergence of the parallel runs was determined by examining the average standard deviation of split frequencies, which fell below 0.01. The first 25% of trees generated were discarded as burn-in and the remaining trees were used to build a majority-rule consensus tree. The ML and Bayesian analyses were separately conducted based on the three plastid genomic regions (LSC, IR, and SSC).

## Results

### *De novo* assembly and annotation of the *Dipteronia* transcriptome

**Figure 1:**
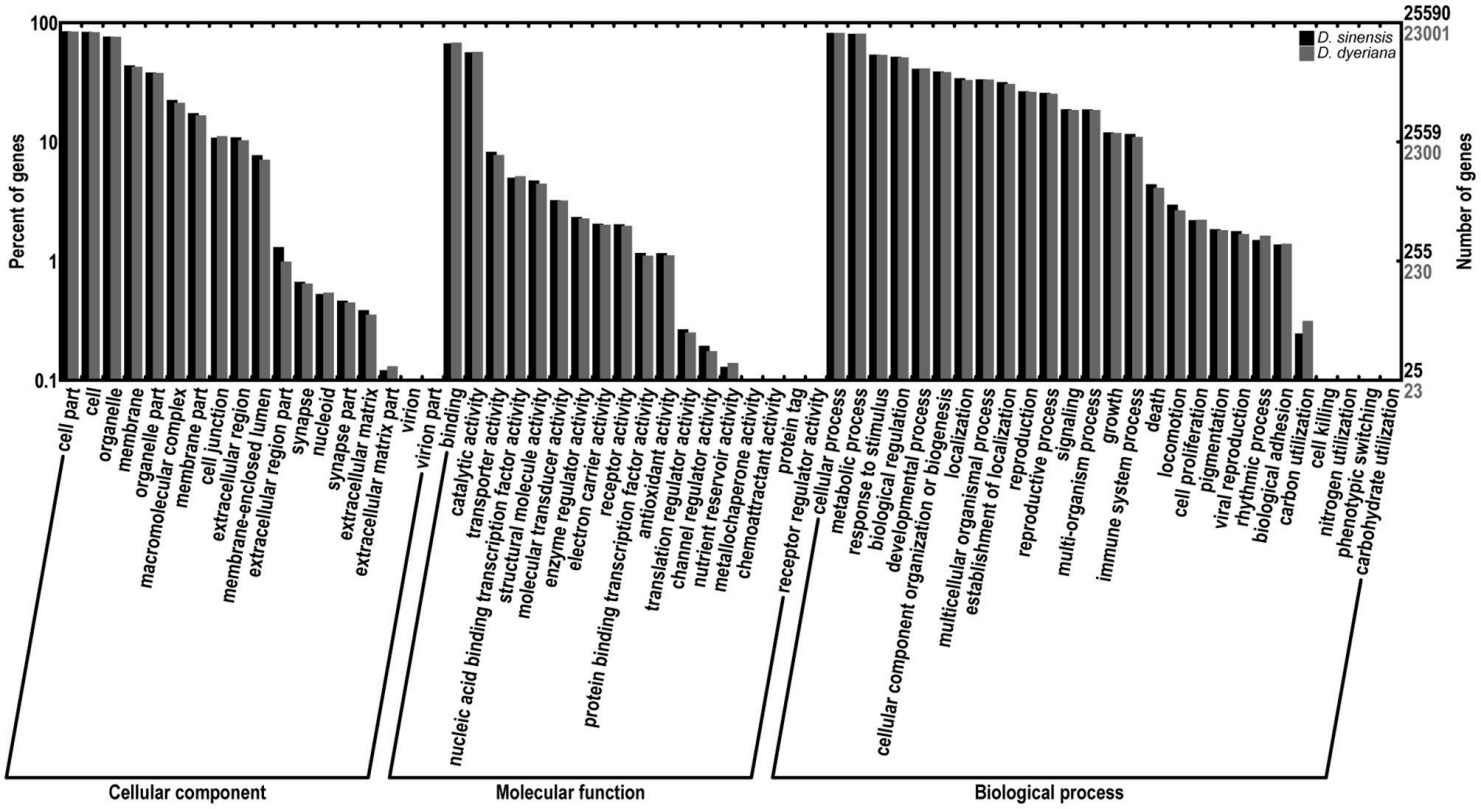
**Comparison of gene ontology (GO) terms distributions between ***D. sinensis*** and ***D. dyeriana*** transcriptome**. GO terms were annotated according to three main categories (biological process, cellular component, molecular function) and 63 sub-categories.

**Figure 2:**
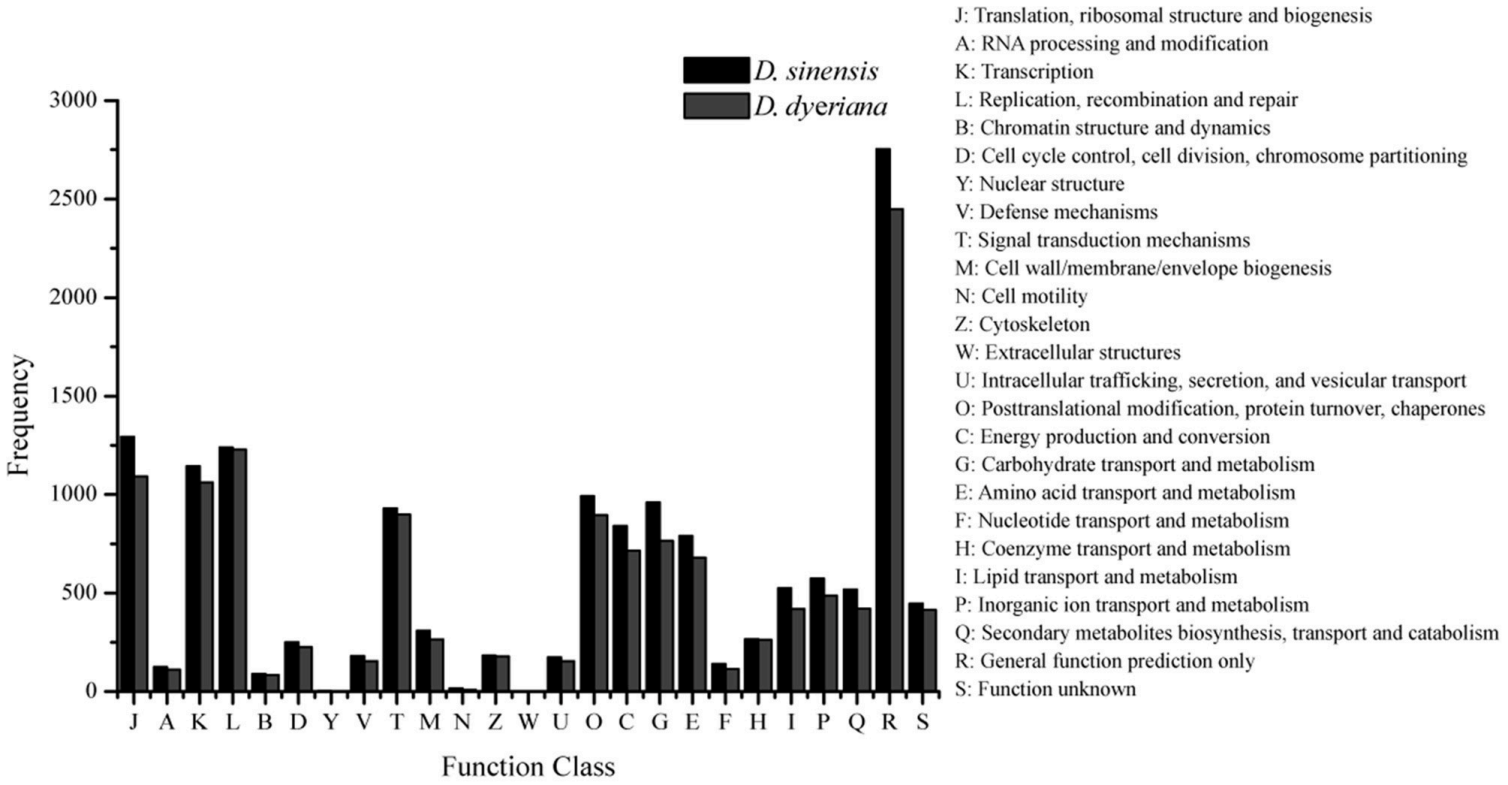
**Clusters of orthologous group (COG) classifications for ***D. sinensis*** and ***D. dyeriana*** transcriptome**. All unigenes were aligned to COG database to predict and classify possible functions.

Using Trinity software, short reads were assembled to generate transcripts, which were further clustered to obtain unigenes. A total of 91,340 transcripts (N50 = 1777 bp, average length = 1055 bp) and 52,351 unigenes (N50 = 1351 bp, average length = 749 bp) were recovered for *D. sinensis*. For *D. dyeriana*, 101,628 transcripts (N50 = 2071 bp, average length = 1248 bp) and 53,983 unigenes (N50 = 1519 bp, average length = 809 bp) were obtained (Table 1). After calculating the FPKM values, our results showed 154/104 unigenes (*D. sinensis*/*D. dyeriana*) with FPKM values >500 (Table S1). To annotate the *D. sinensis* and *D. dyeriana* sequences, searches were conducted against the Nr, Swiss-Prot, COG, KEGG, and GO databases. There were 30,834 unigenes (58.9%) for *D. sinensis* and 27,796 (51.5%) for *D. dyeriana* with at least one significant match to the above databases (Table 2). For Nr annotation of both species, a BLASTX top-hit species distribution showed highest homology to *T. cacao* (8049 hits in *D. sinensis*/7863 hits in *D. dyeriana*) followed by *Vitis vinifera* (3901/3850) and *P. trichocarpa* (2741/2606). GO terms were assigned to 25,591 annotated sequences from *D. sinensis* and 23,003 annotated sequences from *D. dyeriana*. The annotated sequences belonged to three GO categories: “cellular component,” “molecular function,” and “biological process” (Figure 1). We found that the assigned gene functions were similarly distributed in both species. In the “cellular component” category, “cell” (20.1%/21.1%) and “cell part” (21.2%/21.3%) was prominent, while in the “molecular function” category “binding” (43.4%/44.0%) and “catalytic activity” (36.7%/36.8%) were overrepresented. In the “biological process” category, “cellular process” (14.2%/14.3%) was most representative followed by “metabolic process” (13.9%/14.1%). All of the *D. sinensis* and *D. dyeriana* unigenes were subjected to functional prediction and classification using the COG database. The unigenes were assigned to 25 COG categories (Figure 2). The category “cluster of general function” represented the largest group (18.6%/18.7%) in both species. The next most represented category was “translation, ribosomal structure, and biogenesis” for *D. sinensis* (8.8%), while for *D. dyeriana*, “replication, recombination and repair” was the next most represented category (9.4%). Only a few unigenes in both species were assigned into the “nuclear structure” category (4 genes for *D. sinensis* and 1 gene for *D. dyeriana*) and no genes were found in either species in the “extracellular structures” category. To identify the biological pathways of these two species, the annotated unigene sequences were mapped to reference pathways in the KEGG database. The results showed that 7182 unigenes from *D. sinensis* mapped to 120 pathways and 6225 *D. dyeriana* unigenes mapped to 118 pathways. Interestingly, the representative pathways were “ribosome” (673 genes/564 genes, ko03010), “oxidative phosphorylation” (300 genes/252 genes, ko00190), and “glycolysis/gluconeogenesis” (281 genes/269 genes, ko00010) in both species (Figure S1). We also searched the highly expressed unigenes in the GO annotation results and found that many of them were involved in functions related to environmental adaption such as “response to salt stress,” “response to cadmium ion,” “defense response,” “response to water deprivation,” “response to high light intensity.”

**Table 1:** **Summary of statistics for the transcriptomes of ***D. sinensis*** and ***D. dyeriana*****.

	***D. sinensis***	***D. dyeriana***
Total number of reads	40,615,432	53,620,610
Total number of transcripts	91,340	101,628
Total number of unigenes	52,351	53,983
Min length length of unigenes (bp)	201	201
Max length length of unigenes (bp)	14,265	14,906
N50 of unigenes (bp)	1351	1519
Mean length of unigenes (bp)	749	809
Mapping rates of unigenes^a^	74.6%	76.8%

a *Mapping rates were generated by mapping clean reads to the assembled unigenes using the Bowtie mode of RSEM 1.2.29*.

**Table 2:** **Annotation information of ***D. sinensis*** and ***D. dyeriana*****.

	***D. sinensis***		***D. dyeriana***	
	**Number**	**Percentage (%)**	**Number**	**Percentage (%)**
COG	10,637	20.32	9411	17.43
GO	25,591	48.88	23,003	42.61
KEGG	7182	13.72	6225	11.53
Swiss-Prot	23,321	44.55	20,936	38.78
Nr	30,689	58.62	27,738	51.38
All	30,834	58.90	27,796	51.49

### Putative orthologs, substitution rates, and single copy nuclear genes in *D. sinensis* and *D. dyeriana*

By utilizing OrthoMCL and InParanoid methods, we obtained an initial set of 9480 and 9190 putative orthologous pairs in *D. sinensis* and *D. dyeriana*, respectively. After comparing the results of both approaches, 7814 orthologs pairs were found to be common to both methods and were used in subsequent analyses. Synonymous (Ks) and nonsynonymous (Ka) substitution rates were calculated for the orthologous unigene pairs. We excluded orthologous pairs that only had either synonymous or nonsynonymous substitutions; this step left 7699 orthologous unigene pairs that could be used for the calculation of Ka/Ks ratios. In order to avoid paralogs in our analyses, we excluded candidate orthologs with a synonymous (Ks) substitution value >0.1, as these may be paralogs (Zhang J. et al., 2013). Finally, a total of 5435 orthologous pairs was selected and used to calculate Ka/Ks ratios (Table S2). Of these orthologs, 283 had a Ka/Ks ratio >1 indicating positive selection, and 857 had a Ka/Ks ratio between 0.5 and 1, indicating weak purifying selection. The annotation information of orthologs which showed a Ka/Ks ratio significantly >1 (*p* < 0.05) indicated that some of these genes were related to “abiotic and biotic stress response,” “metabolism,” and “enzyme” (Table S3). Using the APVO gene sets (Duarte et al., 2010) to implement BLASTP queries against the 7814 orthologs. Three hundred and fourteen of the APVO genes were found to give hits against orthologous unigenes between *D. sinensis* and *D. dyeriana*; these are most likely the SCNGs of *Dipteronia* species. A total of 54 pairs of orthologs were extracted with more than 600 bp length and >75% identity to *Arabidopsis thaliana* peptide sequences (Table S4).

### Chloroplast genome sequencing, assembly, and annotation

**Figure 3:**
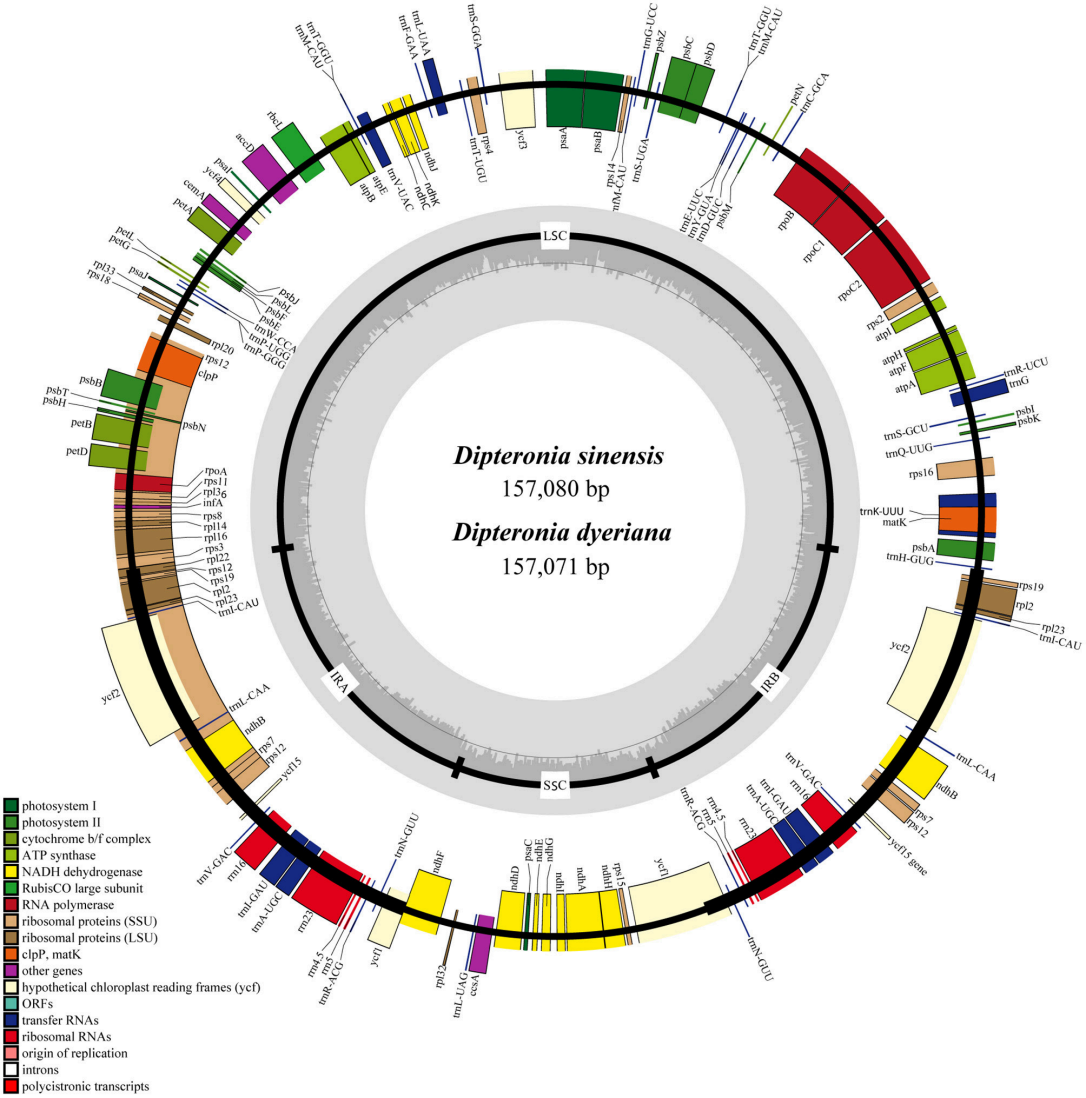
**Circular gene map of ***D. sinensis*** and ***D. dyeriana*** plastomes**. The genes lying outside of the outer circle are transcribed clockwise, while those inside the circle are transcribed counterclockwise. Small single copy (SSC), large single copy (LSC), and inverted repeats (IRa, IRb) are indicated.

Illumina pair-end sequencing produced 25,566,606 and 29,304,216 raw reads with a sequence length of 125 bp for *D. sinensis* and *D. dyeriana*, respectively. The total length of the reads was approximately 7.38 Gb for *D. sinensis* and 6.3 Gb for *D. dyeriana*. After quality trimming of the raw reads, 25,562,204 and 29,221,800 clean reads were collected for *D. sinensis* and *D. dyeriana*, respectively. Based on a combination of *de novo* and reference guided assembly, the complete plastid nucleotide sequences for the two species were recovered. The final chloroplast genome sequences have been deposited in GenBank (Accession numbers: KT878501 and KT985457). The *D. sinensis* and *D. dyeriana* chloroplast genomes were composed of 157,080 bp and 157,071 bp, respectively (Table 3). After annotation, a total of 135 unique genes included 87 protein coding genes, 40 tRNAs, and 8 rRNA operons were obtained for both species (Table S5). The gene map of both species is shown in Figure 3.

**Table 3:** **Summary of two complete chloroplast genomes of ***Dipteronia*****.

	***D. sinensis***	***D. dyeriana***
Total cp DNA size (bp)	157,080	157,071
Length of large single copy (LSC) region (bp)	85,455	85,529
Length of inverted repeat (IR) region (bp)	26,766	26,730
Length of small single copy (SSC) region (bp)	18,093	18,082
Total GC content (%)	37.8	38.0
LSC	35.9	36.1
IR	42.7	42.8
SSC	32.1	32.5
Total number of genes	135	135
Protein encodinga	87 (8)	87 (8)
tRNAa	40 (7)	40 (7)
rRNA^a^	8 (4)	8 (4)

a *The numbers in parenthesis indicate the genes duplicated in the IR regions*.

### Comparative analyses of chloroplast genomes of *Dipteronia*

**Figure 4:**
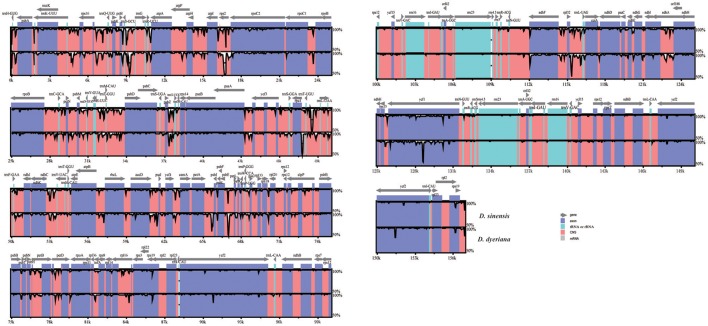
**mVISTA percent identity plot comparing the two ***Dipteronia*** plastid genomes with ***Acer buergerianum*** subsp. ***Ningpoense*** as a reference**. The top line shows genes in order (transcriptional direction indicated by arrows). The sequence similarity of the aligned regions between *Dipteronia* species and *Acer buergerianum* subsp. *Ningpoense* is shown as horizontal bars indicating the average percent identity between 50 and 100% (shown on the y-axis of the graph). The x-axis represents the coordinate in the chloroplast genome. Genome regions are color coded as protein-coding (exon), tRNA or rRNA, and conserved noncoding sequences (CNS).

Both chloroplast genomes exhibited a typical quadripartite structure, consisting of a pair of IRs (26,766 bp in *D. sinensis*/26,730 bp in *D. dyeriana*) separated by an LSC (85,455 bp/85,529 bp) and an SSC (18,093 bp/18,082 bp); there was no significant difference in the lengths of the three regions in the two species. The two chloroplast genomes shared identical complements of genes with similar orders. The GC content of *D. sinensis* and *D. dyeriana* were similar (37.8%/38.0%) (Table 3). The two genomes encode an identical set of 135 genes and 19 are duplicated in the IR regions (Table 3). Of these 135 genes, 15 genes (*rpl2, ndhB, trnI-GAU, trnA-UGC, ndhA, rpl16, petD, petB, trnV-UAC, trnL-UAA, rpoC1, atpF, trnG, rps16, trnK-UUU*) harbored one intron and three genes (*clpP, rps12, ycf3*) harbored two introns (Table S5). Two genes (*infA, rps2*) were inferred to be pseudogenes in *A. buergerianum* subsp. *Ningpoense* (Yang J. B. et al., 2014). The sequence identity of the two *Dipteronia* chloroplast genomes was plotted with mVISTA software (Figure 4). The whole aligned chloroplast genome sequences indicated that they were relatively conserved in the two *Dipteronia* species and *A. buergerianum*, although some highly divergent regions were found. Similar to most plant species, the chloroplast gene coding regions were more conserved than those of their noncoding counterparts. According to the alignment results, several intergenic regions were found to display high divergence, including *trnS*(*GCU*)-*trnG, trnT(UGU)-rps4, trnL(UAA)-trnT(UGU), psbE*-*petL*, and rpl32-*trnL*(*UAG*). Additionally, we found that the level of variation in the noncoding regions (1.96%) was 2.5-fold greater than that in the coding regions (0.79%) and that the IRs and coding regions were more conserved than single copy and noncoding regions, respectively (Figure S2).

Analyses of repeat sequences in the genomes using the REPuter program showed that the characteristics of repeat sequences were similar in the two genomes: 27 repeats were >30 bp in *D. sinensis* and 28 repeats were >30 bp in *D. dyeriana*. Using the Tandem Repeats Finder program, 11 and 15 tandem repeats were identified in *D. sinensis* and *D. dyeriana*, respectively (Tables S6, S7). Most of the repeats were distributed in intergenic (IGS) or intronic regions; a few were located in genic regions (*psaA, psaB*, rps2, rps19, *ycf1, ycf2, trnS-GCU, trnS-UGA, trnS-GGA*) (Tables S6, S7). A total of 118 and 80 microsatellite loci were detected in *D. sinensis* and *D. dyeriana* chloroplast genomes, respectively. The most abundant repeat type in both genomes was mononucleotide repeats (Figure S3). In order to investigate the evolutionary characteristics of cpDNA genes, nonsynonymous (Ka) and synonymous substitution rates (Ks), and the ratio Ka/Ks were calculated for the 87 individual protein coding genes in the two species. The Ka values ranged from 0 to 0.08, the Ks values ranged from 0.007 to 0.03, and most Ka/Ks ratios were less than 1, suggesting that cpDNA genes were under purifying selection. Only four genes (*rpl32, rpl22, rpl33, cemA*) had Ka/Ks ≥ 1 indicating that they had undergone positive selection or neutral selection (Table S8).

### Phylogenetic analyses based on the complete chloroplast genome

**Figure 5:**
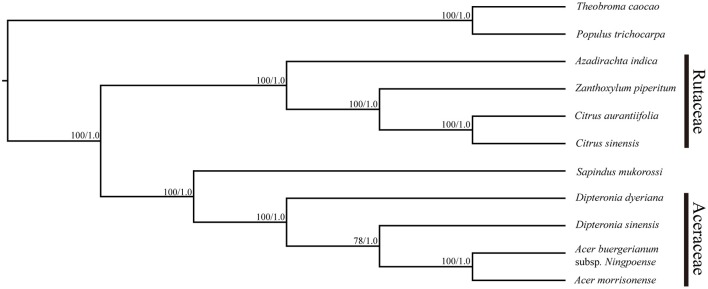
**Maximum likelihood phylogeny of the nine Sapindales species based on the complete plastid genome sequences**. The numbers associated with the nodes are bootstrap support and posterior probability values.

The plastid genomes (with one IR region removed) of 11 species, including *D. sinensis* and *D. dyeriana*, were used to construct a phylogenetic tree. The data set comprised of 152,721 nucleotide positions with 10,179 informative sites for the ingroup taxa. However, there were only 458 informative sites for the four aceraceous species. ML analyses resulted in a fully resolved tree with 9 of the 10 nodes supported by 100% bootstrap values; all the species of Aceraceae formed a monophyletic clade (Figure 5). With respect to the Bayesian analysis, the identical topology was obtained with a posterior probability of 1.0. ML and Bayesian analyses were separately conducted using the LSC, IR, and SSC genomic regions; these analyses yielded an identical topology with all aceraceous species in a monophyletic clade (Figures S4–S6). The two *Dipteronia* species did not cluster in the same clade except when the SSC region was used to construct the phylogenetic tree, indicating that there is considerable divergence between *D. sinensis* and *D. dyeriana*.

## Discussion

### Transcriptome sequencing, *De novo* assembly, and annotation for *D. sinensis* and *D. dyeriana*

Illumina-based transcriptome sequencing has been proven to be an efficient and cost-effective way to retrieve transcriptome data. Recently, many assembled transcriptomes of non-model species have been obtained and employed for studies of differential gene expression, genetic marker development (Huang et al., 2015), and phylogenomic analysis (Yang X. et al., 2014), as well as for detecting selection and inferring adaptive evolution in closely related species (Chen et al., 2015; Guo et al., 2016). To date, however, most transcriptome studies have been carried out on single species. Here, 40.6 million and 53.6 million clean reads were assembled into 52,351 unigenes with a mean length 749 bp for *D. sinensis* and 53,983 unigenes with a mean length of 809 bp for *D. dyeriana*. These results are comparable to those reported previously using the same technology (Li S. S. et al., 2015; Rong et al., 2016). Therefore, the transcriptome datasets produced in the present study will boost the previously meager genomic resources for Aceraceae species.

More than half of the unigenes of both species (58.9%/51.5%) could be annotated using five public protein databases and most involved plant proteins. However, a significant number of unigenes had no BLAST hits to these databases. This may be because there are no comprehensive genomic resources for *Dipteronia* and also because of the lack of a reference genome for Aceraceae; these unigene sequences might therefore represent novel transcripts. Comparative analyses of the functional annotation for the two species showed that they had a similar distribution of functional categories in different protein databases. This may be due in part to use of the same tissues from both species for transcriptome sequencing; alternatively, there may be no significant differences in the protein coding genes of the two species. Intriguingly, a higher number of unigenes were obtained for *D. dyeriana*, although the number of annotated unigenes for *D. sinensis* was greater than for *D. dyeriana*. This difference suggested that the unigene sequences of *D. dyeriana* might include a greater proportion of novel transcripts. Highly expressed genes in both species did not show identical functions, although most of these genes were involved in functions related to environmental adaption. We presume that the different habitat preferences of the two species stimulated this genetic divergence.

### Orthologous genes, substitution rates, and SCNGs markers in *D. sinensis* and *D. dyeriana*

Ka/Ks values are widely used to distinguish protein coding genes under positive or purifying selection (Hurst, 2002). Orthologs under positive selection contain interesting candidate genes that are usually related to “abiotic and biotic stress response,” “biosynthesis,” and “metabolism and enzyme” (Zhao et al., 2013). In the present study, 5435 orthologous pairs were analyzed and 30 were found to have a Ka/Ks ratio significantly >1; some of these orthologs were related to the above-mentioned functions such as “response to stress” (GO:0006950), “response to salt stress” (GO:0009651), “metabolic process” (GO:0008152), and “oxidative stress” (GO:0034599). We thus deduced that such genes suffered significant positive selection during evolution; these results are in line with those reported in previous studies on non-model species (Zhang J. et al., 2013; Zhang L. et al., 2013; Zhao et al., 2013). One orthologous pair was found to be a member of the subtilase protein family which is involved in seed coat development (GO:0048359) (Rautengarten et al., 2008). Therefore, we infer that these genes were also under significant positive selection and would result in differences in seed characters in the two species. Additionally, some orthologs were detected and annotated with a function in response to UV (GO:0071492). *D. dyeriana* is generally found in locations at comparatively high altitudes; we speculated that this species is subject to more intense ultraviolet light exposure that might affect expression of genes related to UV response. The remaining 5151 orthologous pairs had a Ka/Ks < 1; 4041 orthologs had a Ka/Ks < 0.5 (*p* < 0.05), suggesting that most genes are likely to undergo purifying selection with stronger selective constraints for nonsynonymous changes than for synonymous ones (Tiffin and Hahn, 2002). If a Ka/Ks ratio >0.5 is considered an indicator of positive selection, as in previous studies (Swanson et al., 2004), then 1140 pairs with a Ka/Ks ratio between 0.5 and 1 were detected. This indicates a large number of orthologous pairs in *D. sinensis* and *D. dyeriana* with a relatively high Ka/Ks value. One factor that increases Ka/Ks value as well as weakening the strength of purifying selection is a decrease in the effective population size (Fay and Wu, 2003). Both *D. sinensis* and *D. dyeriana* are listed as endangered Tertiary relic species. Thus, in our study, reduced effective population sizes may have contributed to the relatively high Ka/Ks ratios.

Previous studies described genetic markers, such as SSRs, in *Dipteronia* (Chen et al., 2011; Su et al., 2012; Zhou et al., 2016) but no SCNGs markers have been developed. SCNGs with heterogeneous rates of variation are generally thought to provide a higher level of discrimination than chloroplast and nuclear ribosomal (nrDNA) spacer sequences (Salas-Leiva et al., 2014; Mao et al., 2016). Recently, single copy or low copy nuclear genes have been increasingly used to clarify phylogenetic relationship in some angiosperms and to determine the dynamics of speciation (Curto et al., 2012; Zhang N. et al., 2012; Du et al., 2015; Guo et al., 2015). Until now, only nrDNA and chloroplast markers have been used to probe phylogenetic relationships between *Dipteronia* and related genera (Yang et al., 2010). The large number of SCNGs developed in the present study will contribute substantially to the elucidation of phylogenetic relationships and to investigation of population demographic history in *Dipteronia* and Aceraceae species.

### Comparative analyses of complete chloroplast genome sequences

The present study produced complete chloroplast genomes for each of the *Dipteronia* species using Illumina sequencing technology. Apart from the plastid genomes of *A. buergerianum* subsp. *Ningpoense* and *A. morrisonense*, no published chloroplast genomes have been reported for Aceraceae. Therefore, our determination of the whole plastid genomes for the two *Dipteronia* species will be a significant aid to filling in the gap in our knowledge of plastid genome evolution in *Dipteronia* and *Acer* species. The two plastid genomes described here possess the typical angiosperm quadripartite structure with two short inverted repeat regions separated by two single copy regions. The size, gene content, and organization of the plastomes of *Dipteronia* are similar to that of *A. buergerianum* subsp. *Ningpoense* and no significant structural rearrangements, such as inversions or gene relocations, were detected. The chloroplast genomes of both species in this study were relatively well conserved, and most variations were detected in intergenic regions; a similar effect was seen in two other species of Aceraceae (Figure 5). One of the aims of this type of study is to identify genomic “barcodes”; these are DNA sequences with a sufficiently high mutation rate to identify a species within a given taxonomic group (Li X. et al., 2015). Here, we found highly variable regions in *accD, rpl33, rpl22, psaC, rps16/trnQ-UGG, trnS(GCU)/trnG-GCC*, and *trnL-UAA/trnF-GAA*; this variation may be sufficient to suggest these are candidate gene regions for developing more specific DNA barcodes for the Aceraceae family. Such variable markers could also be used to further clarify phylogenetic relationships in aceraceous plants.

As repeat elements are correlated with plastome rearrangement (Weng et al., 2013), we decided to investigate the large, tandem, dispersed, and palindromic repeat sequences in the plastomes of *Dipteronia*. We identified a similarly low number of repeats in the two chloroplast genomes; these repeats were usually located in the same genes (*ycf1, ycf2*) or in genes with similar functions (*psaA, psaB*; *trnS-GCU, trnS-UGA, trnS-GGA*) in both species. Low numbers of repeats have also been found in other species of Geraniaceae and Chloridoideae (Weng et al., 2013; Rousseau-Gueutin et al., 2015). Additionally, SSRs were also distributed similarly in two chloroplast genomes and most of these were located in the same regions of both genomes. For protein coding genes in both species, sequence divergence was evaluated by comparing the synonymous (Ks) substitution rates; all of the genes showed a low sequence divergence (Ks < 0.1) except for *psaC* (Ks = 0.114). For all protein coding genes, most Ka/Ks value were < 1 which indicated that most chloroplast genes were under purifying selection; this is consistent with previous studies (Rousseau-Gueutin et al., 2015; Xu et al., 2015). Only three genes (*rpl32, rpl22, cemA*) had a Ka/Ks ratio >1 as expected of genes under positive selection. Of these genes, *rpl32* and *rpl22* encoded ribosomal proteins. A previous study also found that ribosomal proteins have more divergent protein sequences than genes for photosynthesis (Xu et al., 2015). Interestingly, the *cemA* gene is related to the PPR7 protein. We speculated that *cemA* may have coevolved with nuclear genes (Jalal et al., 2015).

### The phylogenetic position of *Dipteronia* chloroplast genome sequences

Plastid genomes have been proven to be effective in resolving difficult phylogenetic relationships (Ma et al., 2014; Carbonell-Caballero et al., 2015). In the present study, 11 complete chloroplast genomes of five taxa were used to resolve the still-debated phylogenetic position of *Dipteronia* species (Yang et al., 2010). In our analyses, all the species of Aceraceae formed a monophyletic clade with a high-resolution value and clustered with *S. mukorossi* (Sapindaceae) in the same clade. This result is compatible with the proposal that the *Dipteronia-Acer* clade is a subfamily (Aceroideae) or lower rank within the Sapindaceae (McClain and Manchester, 2001). Although traditional plant taxonomy considers *Dipteronia* and *Acer* as sister taxa, *D. sinensis* and *D. dyeriana* were not clustered into a monophyletic clade and did not show a paraphyletic relationship with *Acer* in the current study. Both the BI and ML analyses showed coincident topology based on different plastid regions (except for the SSC) and this was used to construct the phylogeny in which *D. sinensis* and *D. dyeriana* were clustered into a monophyletic clade. The phylogenetic trees based on complete chloroplast genomes and three different plastid regions in this study indicated that *D. sinensis* is usually in parallel with *Acer* but not with *D. dyeriana*, as was suggested in a previous study (Yang et al., 2010). This significant discrepancy in phylogenetic placement of *D. dyeriana* should be interpreted with caution. First, only a few chloroplast gene fragments were utilized in the previous study to construct the phylogenetic relationships. Since phylogenomics has been proven a robust method for tackling difficult phylogenies, the results of the present study may therefore provide a more reliable conclusion (Bewick et al., 2012; Zhou et al., 2012; Ma et al., 2014; Carbonell-Caballero et al., 2015). Second, as tertiary species with allopatric distribution ranges have undergone a long-term complex evolutionary history, involving different geological and climate events over a long period, this may be the cause of the high genetic divergence between *D. sinensis* and *D. dyeriana*. Finally, as *D. sinensis* is present in a wide range of natural habitats and is sometimes located in the same areas as *Acer* plants, it is possible that there might have been hybridization events between *D. sinensis* and *Acer* species during the evolutionary process, which may have significantly affected its phylogenetic position. Determination of whether *D. sinensis* is always in parallel with *Acer* will require analysis of more *Acer* chloroplast genomes in future. Overall, our analysis of chloroplast genomes has provided a valuable resource for future work on the phylogenetics of *Dipteronia* species.

## Author contributions

GZ and TZ conceived and designed the experiments. TZ, CC, YW, and YC performed the experiments and analyzed the data. GB prepared the samples. TZ wrote the paper. ZL and NK help to revise the paper. All authors read and approved the final manuscript.

### Conflict of interest statement

The authors declare that the research was conducted in the absence of any commercial or financial relationships that could be construed as a potential conflict of interest.

## Supplementary material

Table S1 **Gene expression level of two *Dipteronia* species**.

Table S2 **Ka, Ks values, and Ka/Ks ratio between orthologs in two *Dipteronia* species**.

Table S3 **Annotation results of 30 orthologous pairs with Ka/Ks > 1 (*p* < 0.05)**.

Table S4 **The list of 55 candidate single copy nuclear genes homologous to APVO genes**.

Table S5 **List of genes present in *Dipteronia* chloroplast genome**.

Table S6 **The repeats distribution in *D. sinensis* chloroplast genome**.

Table S7 **The repeats distribution in *D. dyeriana* chloroplast genome**.

Table S8 **Ka/Ks ratio between pairwise of species protein coding sequences in two *Dipteronia* species**.

Table S9 **List of primer pairs used in sequence verification and improvement of the *Dipteronia* chloroplast genome**.

Figure S1 **Kyoto Encyclopedia of Genes and Genomes (KEGG) classification between *D. sinensis* and *D. dyeriana* transcriptome**.

Figure S2 **Percentage of variable characters in aligned two *Dipteronia* chloroplast genomes. (A)** Coding region. **(B)** Noncoding region. These regions are oriented according to their locations in the chloroplast genome.

Figure S3 **Frequency distribution of the SSRs identified in *Dipteronia* plastid genomes**.

Figure S4 **Maximum likelihood phylogeny of the nine Sapindales species based on the large single copy (LSC) region sequences**. The numbers associated with the nodes are bootstrap support and posterior probability values.

Figure S5 **Maximum likelihood phylogeny of the nine Sapindales species based on the inverted repeat A (IRa) region sequences**. The numbers associated with the nodes are bootstrap support and posterior probability values.

Figure S6 **Maximum likelihood phylogeny of the nine Sapindales species based on the small single copy (SSC) region sequences**. The numbers associated with the nodes are bootstrap support and posterior probability values.
