# Supplementary figures and images for: Comparative Transcriptome and Chloroplast Genome Analyses of Two Related Dipteronia Species
Source: Front Plant Sci. 2016 Oct 13;7:1512. doi: 10.3389/fpls.2016.01512 (PMC5061820; doi:10.3389/fpls.2016.01512)

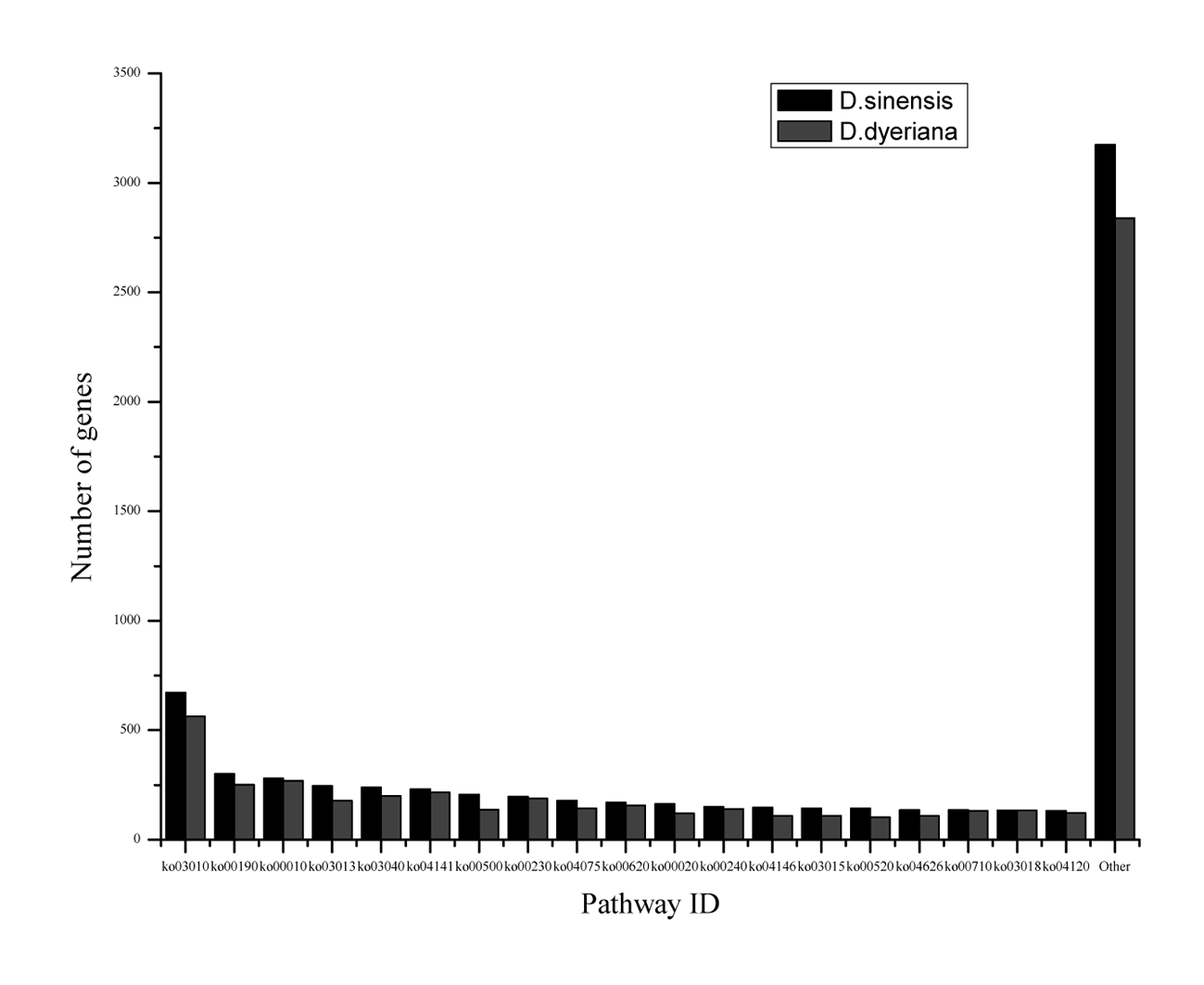

Supplement: Figure S1 — Kyoto Encyclopedia of Genes and Genomes (KEGG) classification between D. sinensis and D. dyeriana transcriptome. [file Image1.TIF]

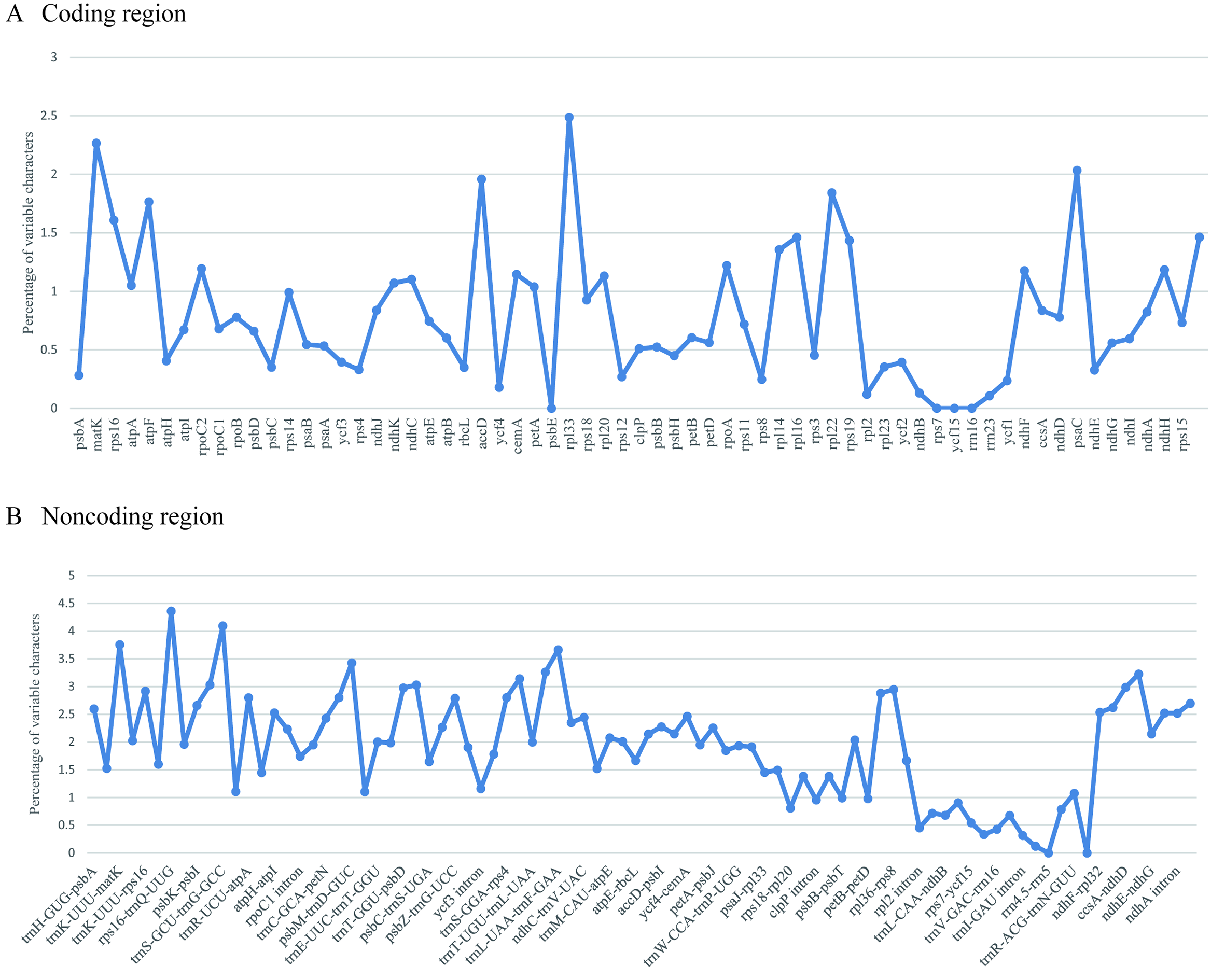

Supplement: Figure S2 — Percentage of variable characters in aligned two Dipteronia chloroplast genomes. (A) Coding region. (B) Noncoding region. These regions are oriented according to their locations in the chloroplast genome. [file Image2.TIF]

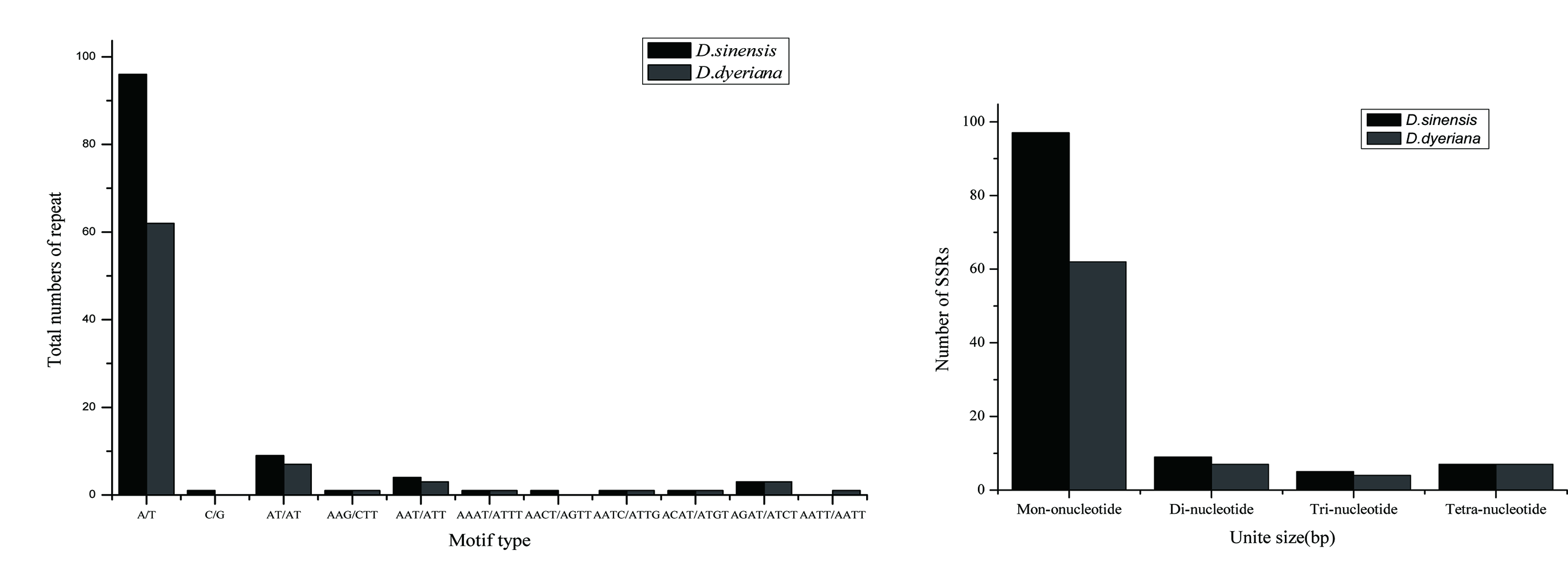

Supplement: Figure S3 — Frequency distribution of the SSRs identified in Dipteronia plastid genomes. [file Image3.TIF]

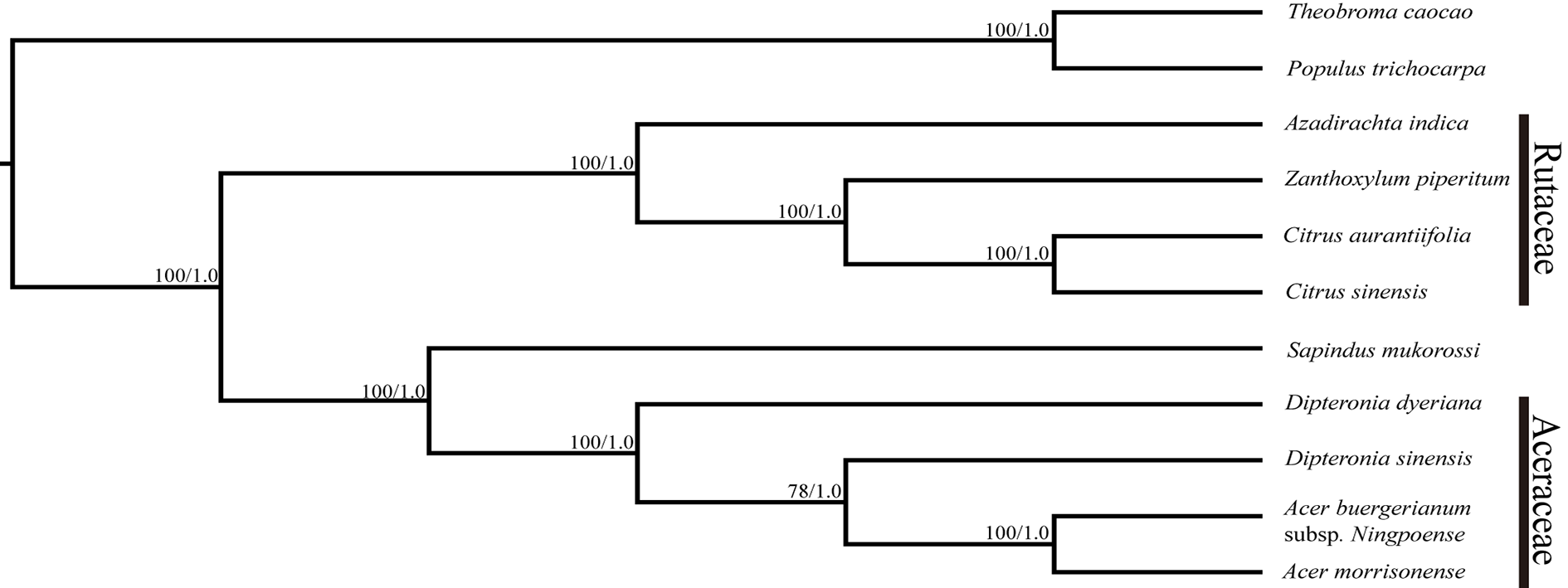

Supplement: Figure S4 — Maximum likelihood phylogeny of the nine Sapindales species based on the large single copy (LSC) region sequences. The numbers associated with the nodes are bootstrap support and posterior probability values. [file Image4.TIF]

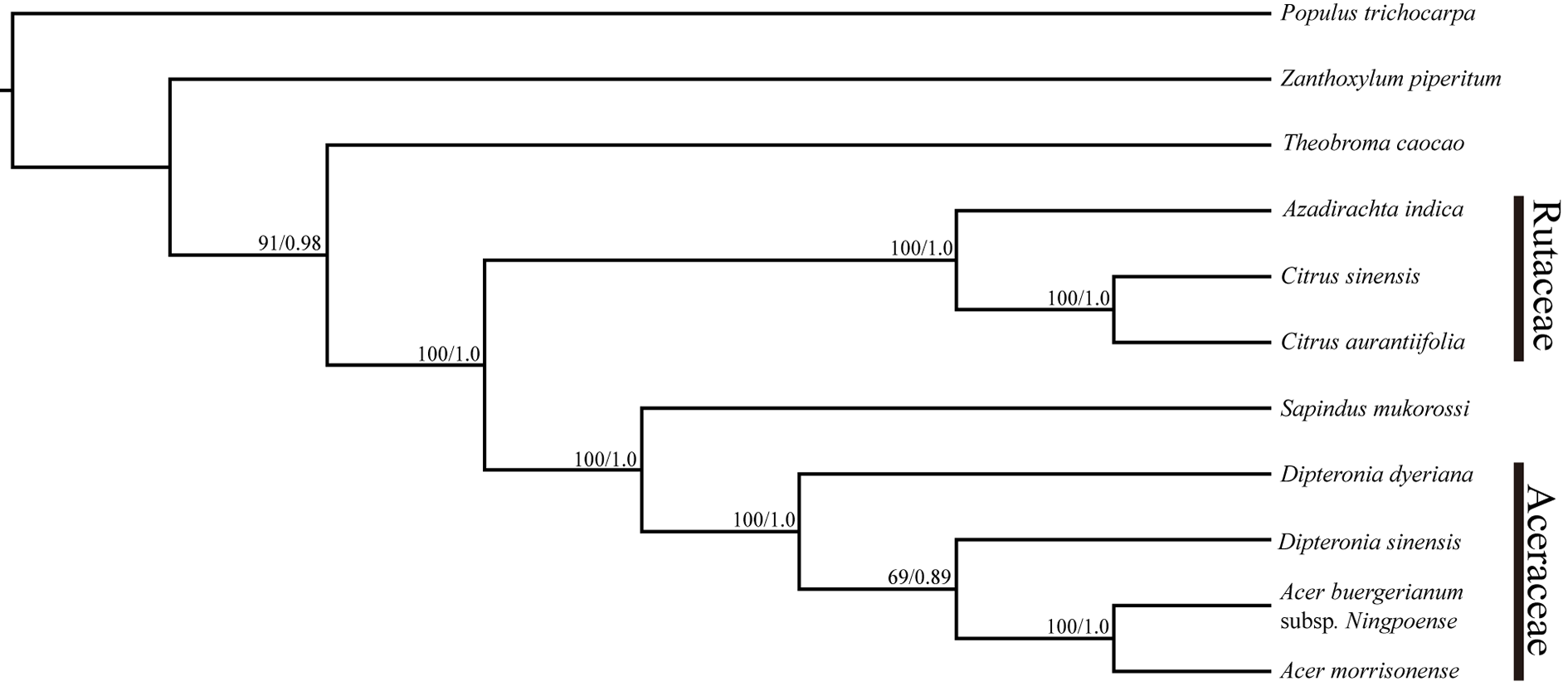

Supplement: Figure S5 — Maximum likelihood phylogeny of the nine Sapindales species based on the inverted repeat A (IRa) region sequences. The numbers associated with the nodes are bootstrap support and posterior probability values. [file Image5.TIF]

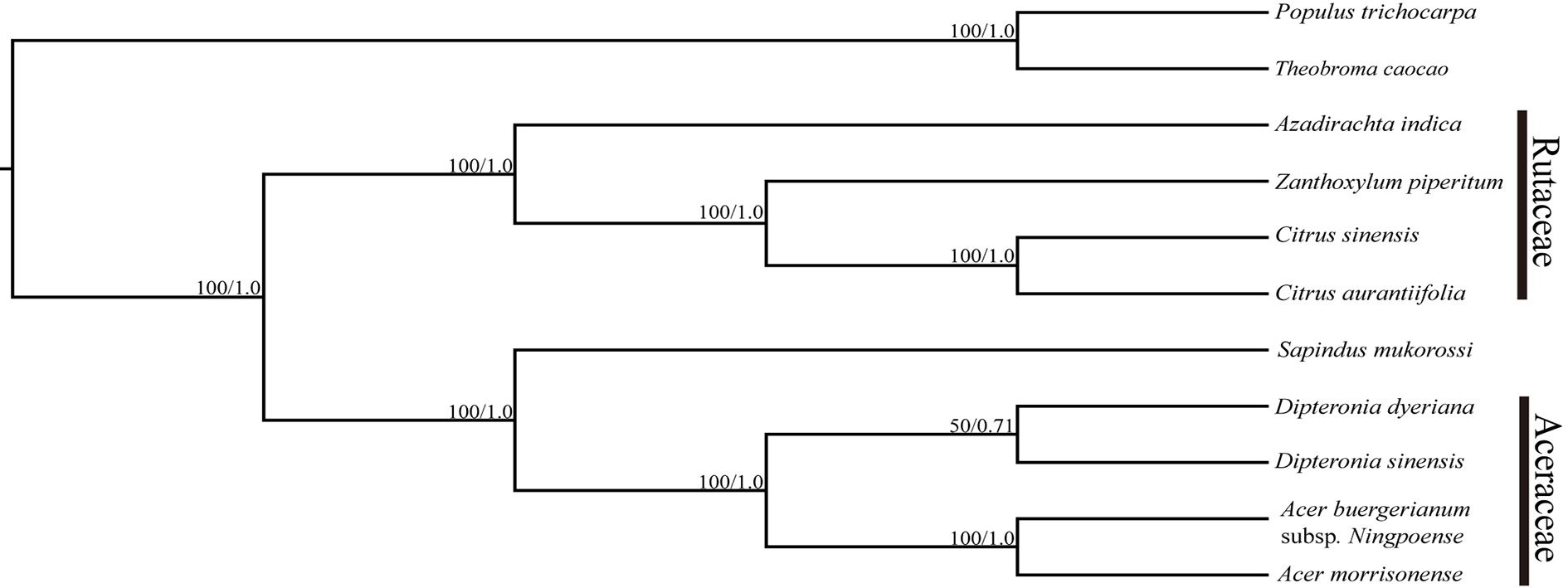

Supplement: Figure S6 — Maximum likelihood phylogeny of the nine Sapindales species based on the small single copy (SSC) region sequences. The numbers associated with the nodes are bootstrap support and posterior probability values. [file Image6.TIF]
